# Myelin oligodendrocyte glycoprotein antibody-associated disease as a novel presentation of central nervous system autoimmunity in a pediatric patient with Wiskott-Aldrich syndrome

**DOI:** 10.1186/s13223-023-00827-x

**Published:** 2023-08-07

**Authors:** Vivien X. Xie, Wilson File, Christina Wiedl, Brant R. Ward, Blachy Dávila Saldaña, Michael D. Keller, Alexandra B. Kornbluh

**Affiliations:** 1 Department of Neurology, District of Columbia, Children’s National Hospital, 111 Michigan Ave NW, Washington, 20010 USA; 2 Division of Hematology and Oncology, Eastern Virginia Medical School and Children’s Hospital of The King’s Daughters, Norfolk, VG USA; 3 Division of Hematology and Oncology, District of Columbia, Children’s National Hospital, Washington, USA; 4 Division of Allergy and Immunology, Children’s Hospital of Richmond, Virginia Commonwealth University, Richmond, VA USA; 5 Division of Blood and Marrow Transplantation, Children’s National Hospital, Washington, DC USA; 6 Center for Cancer and Immunology Research, Division of Allergy and Immunology, Children’s National Hospital, Washington, DC USA

**Keywords:** Wiskott-Aldrich syndrome, Optic neuritis, Demyelination, Myelin oligodendrocyte glycoprotein antibody associated disease

## Abstract

**Background:**

Wiskott-Aldrich syndrome (WAS) is an X-linked primary immunodeficiency caused by mutations in the *WAS* gene that leads to increased susceptibility to infections, thrombocytopenia, eczema, malignancies, and autoimmunity. Central nervous system (CNS) autoimmune manifestations are uncommon.

**Case Presentation:**

We describe the case of a five-year-old boy with refractory thrombocytopenia and iron deficiency anemia who developed relapsing bilateral optic neuritis. Myelin oligodendrocyte glycoprotein antibody (MOG-IgG) via serum fluorescence-activated cell sorting assay was positive (titer 1:100), confirming a diagnosis of myelin oligodendrocyte glycoprotein antibody-associated disease (MOGAD). At age six, molecular panel testing for genes associated with primary immunodeficiency identified a missense *WAS* gene variant. He was subsequently found to have decreased WAS protein expression, consistent with a diagnosis of WAS.

**Conclusions:**

This case expands the reported spectrum of CNS autoimmunity associated with WAS and may help to inform long-term therapeutic options.

## Background

Wiskott-Aldrich syndrome (WAS) is an X-linked primary immunodeficiency caused by mutations in the *WAS* gene that leads to increased susceptibility to infections, thrombocytopenia, eczema, malignancies, and autoimmunity [1]. Only few neurological autoimmune complications of WAS have been described; optic neuritis, central nervous system (CNS) demyelination, and myelin oligodendrocyte glycoprotein antibody-associated disease (MOGAD) have not been previously reported.

MOGAD is an acquired neuroinflammatory demyelinating disorder mediated by autoantibodies against the protein myelin oligodendrocyte glycoprotein (MOG), which is located on the outermost surface of the myelin sheath in the CNS [2]. We describe a case of pediatric MOGAD presenting with relapsing bilateral optic neuritis in a five-year-old boy with WAS.

## Case Presentation

A five-year-old boy with a history of chronic thrombocytopenia, anemia, and focal epilepsy acutely developed binocular vision loss. He was found to be thrombocytopenic at nine months when he presented with conjunctival bleeding and was found to have platelets of 12,000/µL. He was initially suspected to have immune thrombocytopenia, so he was treated with IVIG which resulted in a temporary increase of platelets to 24,000/uL. Bone marrow biopsy was completed and found to be normal. Shortly thereafter he also developed a mild anemia that was thought to be secondary to iron deficiency, and he was started on iron supplementation. Over the next three years he received treatments for thrombocytopenia including repeated infusions of IVIG, corticosteroids (both oral prednisone and several courses of intravenous solumedrol), and mycophenolate. His treatment courses were often fragmented due to inconsistent follow-up, but overall trends of his bloodwork showed that his hemoglobin recovered and platelet count remained low. At age three he was started on eltrombopag, resulting in an increase of platelets to the 40,000–50,000/µL range, though this treatment was interrupted due to elevated transaminases (AST 182 IU/L, ALT of 198 IU/L), a known side effect of the medication.

Regarding his history of epilepsy, he initially presented at two years old with a staring episode suspicious for first-time seizure. One month later he developed generalized convulsive status epilepticus and was subsequently started on levetiracetam 20 mg/kg/day, which he has continued since that time without recurrent seizures.

**Fig. 1:**
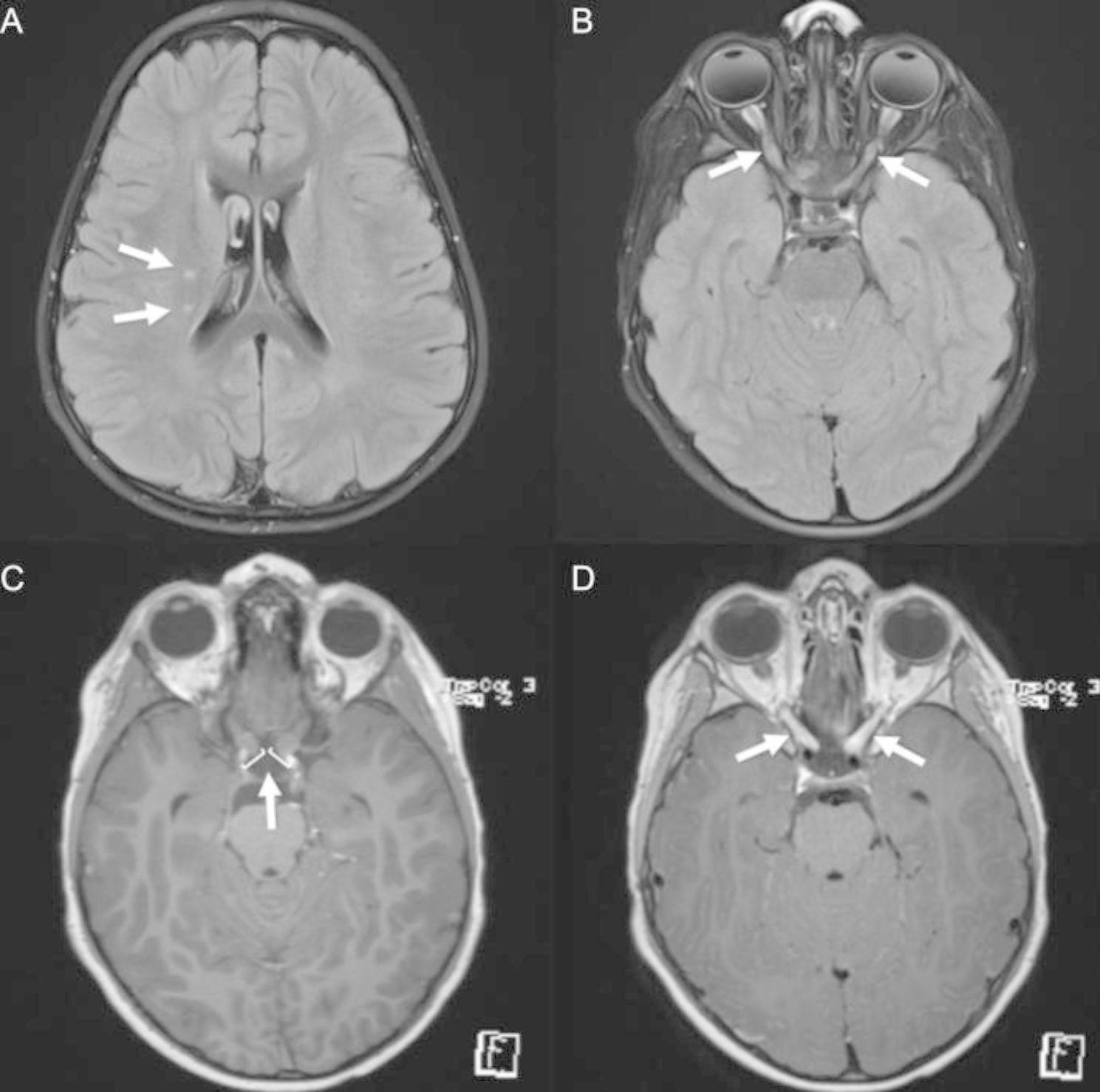
MRI brain images from initial hospitalization. Axial fluid-attenuated inversion recovery (FLAIR) sequences demonstrated **(A)** two small T2/FLAIR hyperintense 3-4 mm foci in the right frontal white matter and **(B)** T2/FLAIR hyperintense bilateral optic nerves. Axial T1 post-gadolinium images demonstrate **(C)** thickening of the optic nerves to 5–7 mm with **(D)** marked enhancement of the nerve sheaths consistent with bilateral optic neuritis

At age five he presented to the hospital after complaining of blurry vision upon waking from sleep. Upon emergency room evaluation his initial neurologic exam was only remarkable for change in visual acuity, with inability to count fingers in both eyes. He was admitted to the hospital and underwent MRI of the brain, which demonstrated enlargement of the bilateral optic nerves associated with marked gadolinium enhancement of the nerve sheaths and two small T2/FLAIR hyperintense foci in the right frontal white matter (Fig. 1). Cerebrospinal fluid (CSF) analysis was unremarkable (white blood cells 3 cells/µL, red blood cells 76 cells/µL, protein 44 mg/dL, and glucose 46 mg/dL). He was treated for suspected optic neuritis with IV methylprednisolone 30 mg/kg daily for 3 days. The patient had good clinical response; on day 3 of steroids his visual acuity was 20/300 in both eyes, and at follow-up it was reported he experienced full recovery of visual acuity. He was discharged with an oral prednisone taper, starting with 60 mg daily and planned for 24 days. His platelet count was noted to transiently improve with corticosteroid treatment, but he restarted eltrombopag as platelets began to decline at reduced steroid dose.

**Fig. 2:**
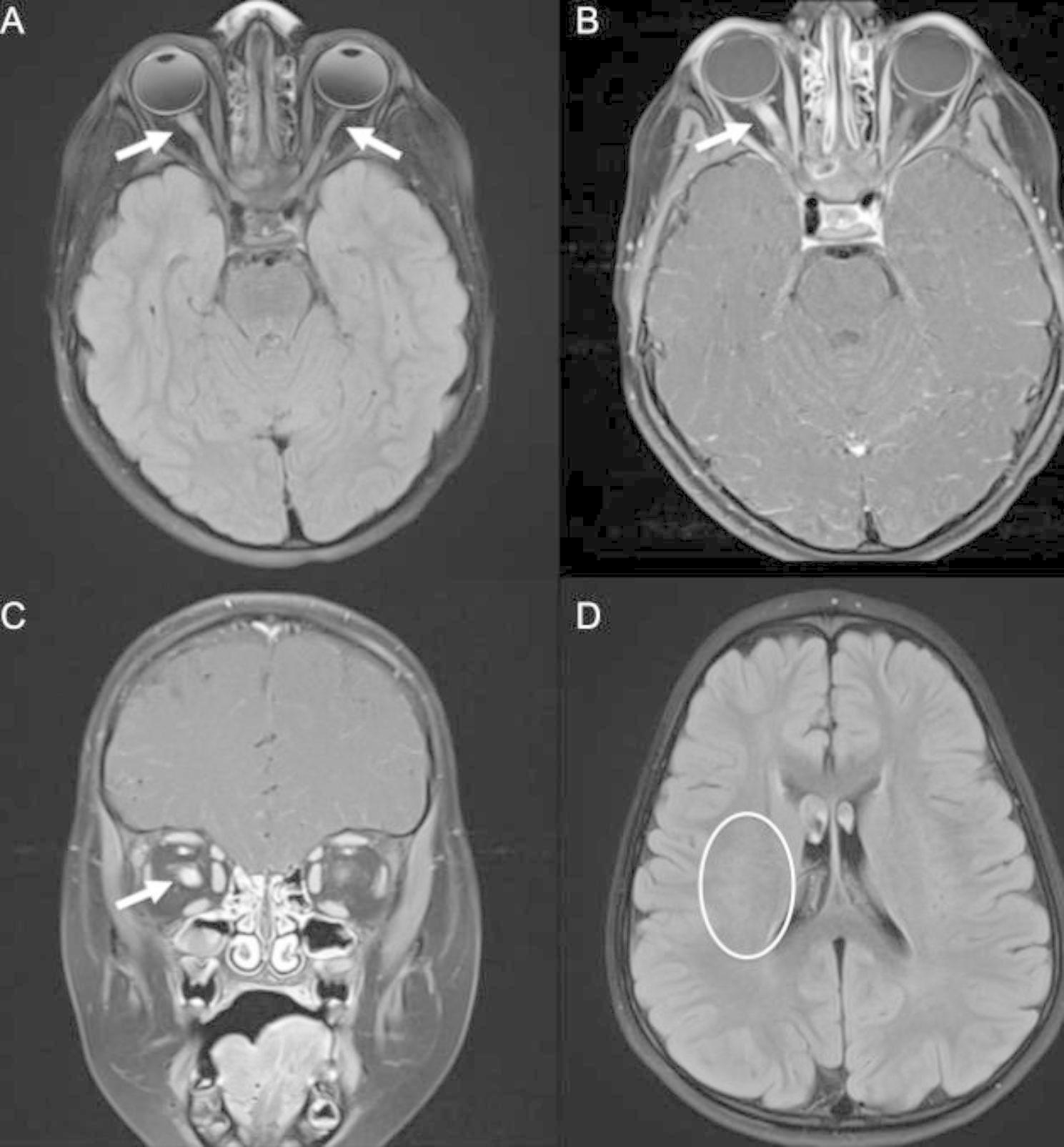
MRI brain images from second hospitalization. Axial FLAIR sequence demonstrating **(A)** T2/FLAIR hyperintensity of bilateral optic nerves right > left, optic chiasm, and optic tracts with **(B)** corresponding T1 gadolinium enhancement, indicating relapsed bilateral optic neuritis. **(C)** T1 gadolinium enhancement of optic nerves right > left also noted in coronal images. **(D)** Previously noted T2/FLAIR hyperintensities resolved

Four months after this hospitalization the patient returned to the emergency room with recurrence of acute onset blurry vision in both eyes. Exam demonstrated afferent pupillary defect of the right eye, and visual acuity testing showed preserved ability to count fingers only in the nasal fields, suspected to indicate blind spot enlargement. MRI of the brain demonstrated enhancement of the bilateral optic nerves (right more than left), expansion and enhancement of the optic chiasm, and resolution of the prior demyelinating lesions (Fig. 2). Due to concern for optic neuritis relapse, he was treated with a five-day course of IV methylprednisolone 30 mg/kg daily. During his hospitalization he experienced moderate improvement of his visual acuity, although he had mild residual deficits in his peripheral vision at time of discharge. In discussion between the patient’s hematologists, immunologists, and neurologists, his eltrombopag was held and he was started on rituximab infusions 375 mg/m^2^ given weekly for four weeks to prevent future recurrences of optic neuritis while potentially serving as treatment for his chronic thrombocytopenia.

In the setting of optic neuritis recurrence, further immunology work-up was initiated while inpatient. He was found to have normal immunoglobulin levels and absent vaccine antibody responses, although it was noted that he had only received two series of childhood immunizations. Lymphocyte enumeration panel was obtained after 2 or 3 doses of high dose methylprednisolone and revealed low absolute B cell (CD19 86 cells/µL, CD20 84 cells/µL), NK cell (CD16 53 cells/µL and CD56 64 cells/µL), and T cell (CD3 126 cells/µL) counts with poor T cell function with absent response to PHA. Following rituximab treatment, he was started on monthly IVIg to prevent rituximab-induced hypogammaglobulinemia and reduce risk of infection. He was also started on trimethoprim/sulfamethoxazole prophylaxis for increased risk of opportunistic infections. Curiously, the patient did not have a history of recurrent infections, which may be related to the family’s persistence in limiting exposures including the decision to home school.

Following discharge, serum MOG-IgG via fluorescence-activated cell sorting assay returned positive with a titer of 1:100, confirming a diagnosis of MOGAD; serum aquaporin 4-IgG was negative. A primary immunodeficiency genetic panel identified a missense mutation in the *WAS* gene (c. 70 C > T, p.Ser24Pro), which has been previously described in another child with WAS [3]. WAS flow cytometry confirmed decreased WASp expression with staining ratio of 0.22 (ref. 0.71–1.31), consistent with a diagnosis of underlying WAS. He has since transitioned from monthly IVIg to treatment with subcutaneous immunoglobulin 180 mg/kg every 2 weeks for hypogammaglobulinemia and eltrombopag 50 mg daily for thrombocytopenia. The genetic panel also identified a variant of uncertain significance in the *AIRE* gene (c.560 C > T, p.Ser187Leu), and subsequent testing for anti-interferon-omega antibodies was negative. A subsequent lymphocyte enumeration panel performed 2 years later continued to demonstrate low T cell count (CD3 585 cells/µL). Hematopoietic stem cell transplantation (HSCT) has been strongly recommended for definitive WAS treatment but has not been undertaken.

The patient was subsequently lost to neurology follow-up for three years after initial presentation with optic neuritis. When he returned to neurology clinic, his exam showed visual acuity of 20/25 in the right eye and 20/25 − 1 in the left eye, and 5 out of 8 properly identified color plates, with an otherwise unremarkable neurologic exam. Repeat brain MRI and serum MOG-IgG have been recommended and results are pending.

## Discussion and Conclusions

We describe the first documented case of MOGAD in a patient with underlying WAS. WAS is commonly associated with autoimmune manifestations, which are important indicators of clinical outcome. The presence of autoimmunity is a predictor of more severe disease in WAS as part of the criteria for maximal rating in the five-point severity WAS score utilized in clinical practice [1], and is considered a strong indicator for HSCT. Autoimmune manifestations in WAS have been reported with variable frequency; several large cohort studies have reported that 9–72% of patients with WAS have autoimmune conditions, including autoimmune hemolytic anemia, thrombocytopenia, vasculitis, and arthritis, among others [4]. There are few reported neurologic manifestations to date. Two published case series describe cerebral vasculitis in five patients aged one to seven years, with one complicated by fatal occlusion of multiple cerebral arteries [5, 6]. Another case report describes a nine-month-old pediatric patient who developed Guillain-Barre syndrome, an immune-mediated polyradiculoneuropathy that leads to flaccid paralysis, who subsequently made a full neurologic recovery [7]. Resolution of autoimmune disease is seen in the vast majority of patients with WAS following successful HSCT [8].

Of note, although initial lymphocyte enumeration results were obtained during a course of high dose methylprednisolone, the extreme degree of lymphopenia and absent response to PHA are highly consistent with WAS, and the consistent lymphopenia on later testing also suggests that this is due to his underlying WAS diagnosis. Our patient’s genetic testing also identified a heterozygous variant in the *AIRE* gene, which is associated with autoimmune polyendocrinopathy, candidiasis, and ectodermal dystrophy (APECED) syndrome. This mutation was not felt to be pathogenic in our patient’s case given that APECED syndrome is typically recessive, and anti-interferon omega antibody testing was negative. Ocular manifestations can occur in about 15–25% of APECED patients in the United States, including keratoconjunctivitis [9]. Several case reports also describe patients with *AIRE* mutations presenting with autoimmune retinopathy [10, 11]. Our patient did not present with these other autoimmune ocular manifestations, and there is no known association between optic neuritis and *AIRE* gene mutations.

The mechanisms predisposing to autoimmunity in WAS are not fully elucidated, though studies have proposed altered B cell tolerance by positive selection of self-reactive B cells [12] as well as abnormalities of T regulatory cell function [13]. One study aimed to investigate the role of WASp in CNS autoimmunity by studying a mouse model of experimental autoimmune encephalomyelitis (EAE), the most commonly used experimental model for autoinflammatory demyelinating diseases of the CNS such as multiple sclerosis [14]. WAS-knockout mice were found to have increased autoreactive T-helper cells against MOG antigen, but in the absence of WASp mice were markedly resistant against developing EAE. The authors concluded that although WASp deficiency does not impair the development of myelin-specific autoreactive T-cells, WASp may be a necessary factor in developing neuroinflammatory disease due to its roles in T-cell transmigration into the CNS as well as CNS microglial activation. Additional molecular and cellular studies are needed to determine the nuanced role of WASp in the development of CNS autoimmunity, including contributory mechanisms in human hosts and the broader spectrum of neuroinflammatory manifestations. To our knowledge, there is no previously known clinical association between WAS and CNS demyelinating disorders such as MOGAD.

MOGAD is an acquired demyelinating syndrome (ADS) characterized by immune-mediated inflammation of the optic nerves, brain, and spinal cord associated with the presence of anti-MOG antibodies. MOGAD is more common in the pediatric population; an estimated 30–50% of ADS presentations in children are attributed to MOGAD [15] compared to 1.2–6.5% in adults [16, 17]. In a descriptive cohort of 683 patients aged 1–82 years with ADS out of Israel, 53 patients (7.7%) were positive for MOG-IgG. 121 of the 683 patients studied were children under 18 years of age, and 23 (19%) of those children were MOG-IgG positive [18]. There is no race or gender predominance in younger children, though there may be a slight female predominance in adolescence and adults. In a UK cohort of 252 anti-MOG antibody positive patients with mean age of onset of 30.1 years, 143 participants (57%) were female [19].

MOGAD presents with a varied and age-dependent spectrum of clinical phenotypes in pediatric patients. Acute disseminated encephalomyelitis (ADEM) occurs more commonly in younger children, whereas optic neuritis, longitudinally extensive transverse myelitis (LETM), and neuromyelitis optica spectrum disorders (NMOSD)-like phenotype are more common in older children and adolescents [20]. Our patient presented with simultaneous bilateral optic neuritis, which is a phenotype more typical in adolescents between 13 and 18 years of age but overall has been reported in greater than 50% of pediatric MOGAD [20]. Other pediatric ADS, such as multiple sclerosis, are less likely to present with age of onset less than eleven years or with bilateral optic nerve involvement [21, 22], making our patient’s clinical phenotype more typical of MOGAD.

In addition to ADEM, the phenotypic spectrum of MOGAD includes additional forms of cerebral cortical and autoimmune encephalitides which can be associated with seizures [20]. Though our patient had seizures prior to his diagnosis of MOGAD, there is no clear evidence that the underlying etiology of his epilepsy is due to demyelination, and he otherwise did not have features suggestive of a cortical encephalitis such as encephalopathy or correlative MRI findings. Of note, there is no established relationship between WAS and epilepsy documented in the literature. There is one case series that reports seizures as a complication of intracranial hemorrhage in two patients with WAS, which was not part of our patient‘s complex history [23].

MOGAD most commonly portends a monophasic disease course though up to 30% of children experience a relapse within five years [2]. Generally, the long-term outcomes of MOGAD are reassuring with most patients experiencing complete or near-complete recovery from acute presentations, though some patients are left with residual deficits [24]. Expert opinion in the literature supports use of corticosteroids as the first-line treatment for acute presentations, and intravenous immunoglobulins (IVIG) or plasma exchange (PLEX) are reserved for more refractory or severe presentations [25]. Our patient had good clinical response to first-line acute therapy with corticosteroids. Regarding the need for maintenance immunotherapy, the Pediatric European Collaborative Expert Consensus on treatment of MOGAD recommends maintenance therapy in patients with a relapsing disease course, but to date evidence-based guidelines or informative clinical trials regarding the choice of agent are lacking [26]. Our patient received rituximab for MOGAD relapse prevention in part due to potential dual benefit for his chronic thrombocytopenia, though retrospective data investigating the efficacy of B-cell depleting agents as maintenance therapy in MOGAD is mixed [15]. Our patient did have a transient rise in his platelet count after rituximab administration and has had no further relapses of MOGAD to date.

There is a lower frequency of coexisting autoimmune conditions in patients with MOGAD when compared to other ADS such as aquaporin-4 positive neuromyelitis optica [27]. It is important to recognize, however, that MOGAD may be a novel manifestation of CNS autoimmunity in WAS because the presence of autoimmunity is indicative of more severe WAS with a potentially worse prognosis. Recognition of the signs and symptoms of MOGAD in patients with WAS may lead to earlier consideration of more definitive treatment strategies such as gene therapy or hematopoietic stem cell transplantation, which has been shown to lead to resolution of previous autoimmunity and decreased incidence of *de novo* autoimmune disease [8]. In conclusion, this case expands the reported spectrum of CNS autoimmunity associated with WAS and may help to inform long term therapeutic options.

## Data Availability

All data generated or analyzed during this study are included in this published article.

## List of abbreviations

WAS: Wiskott-Aldrich syndrome
MRI: Magnetic resonance imaging
LP: Lumbar puncture
ON: Optic neuritis
MOGAD: Myelin oligodendrocyte glycoprotein antibody-associated disease
CNS: Central nervous system
CSF: Cerebrospinal fluid
FLAIR: Fluid-attenuated inversion recovery
ADS: Acquired demyelinating syndrome
WASp: Wiskott Aldrich syndrome protein
IVIG: Intravenous immunoglobulin
PLEX: Plasma exchange
